# Cell Cycle Regulation and Cytoskeletal Remodelling Are Critical Processes in the Nutritional Programming of Embryonic Development

**DOI:** 10.1371/journal.pone.0023189

**Published:** 2011-08-17

**Authors:** Angelina Swali, Sarah McMullen, Helen Hayes, Lorraine Gambling, Harry J. McArdle, Simon C. Langley-Evans

**Affiliations:** 1 School of Biosciences, University of Nottingham, Sutton Bonington, Loughborough, United Kingdom; 2 Rowett Institute of Nutrition and Health, University of Aberdeen, Aberdeen, United Kingdom; New Mexico State University, United States of America

## Abstract

Many mechanisms purport to explain how nutritional signals during early development are manifested as disease in the adult offspring. While these describe processes leading from nutritional insult to development of the actual pathology, the initial underlying *cause* of the programming effect remains elusive. To establish the primary drivers of programming, this study aimed to capture embryonic gene and protein changes in the whole embryo at the time of nutritional insult rather than downstream phenotypic effects. By using a cross-over design of two well established models of maternal protein and iron restriction we aimed to identify putative common “gatekeepers” which may drive nutritional programming.

Both protein and iron deficiency *in utero* reduced the nephron complement in adult male Wistar and Rowett Hooded Lister rats (P<0.05). This occurred in the absence of damage to the glomerular ultrastructure. Microarray, proteomic and pathway analyses identified diet-specific and strain-specific gatekeeper genes, proteins and processes which shared a common association with the regulation of the cell cycle, especially the G1/S and G2/M checkpoints, and cytoskeletal remodelling. A cell cycle-specific PCR array confirmed the down-regulation of cyclins with protein restriction and the up-regulation of apoptotic genes with iron deficiency.

The timing and experimental design of this study have been carefully controlled to isolate the common molecular mechanisms which may initiate the sequelae of events involved in nutritional programming of embryonic development. We propose that despite differences in the individual genes and proteins affected in each strain and with each diet, the general response to nutrient deficiency *in utero* is perturbation of the cell cycle, at the level of interaction with the cytoskeleton and the mitotic checkpoints, thereby diminishing control over the integrity of DNA which is allowed to replicate. These findings offer novel insight into the primary causes and mechanisms leading to the pathologies which have been identified by previous programming studies.

## Introduction

Human epidemiological studies have offered an array of evidence to support the theory that major disease states of adulthood are established *in utero* [1], [2], [3]. Early life programming is the process through which insults, such as poor nutrient supply, during the vulnerable periods of gestational and neonatal development, exert permanent effects on organ development, physiology and metabolism. The adaptations to insult are proposed to increase the risk of developing non-communicable diseases in later life. Whilst a range of human cohort studies have demonstrated associations between early life events and later disease risk, they are limited in their ability to demonstrate causality and cannot be used to investigate mechanisms, due to the impact of bias and confounding factors, and the lack of invasive measurements.

Animal models have consequently played an important role in developing understanding of the processes underlying the programming theory, as they allow specific hypotheses to be tested during the critical windows of interest. A variety of small and large models have provided robust evidence to support an association between developmental insults and metabolic risk factors in later life. Rodent models in particular have covered a wide range of nutritional early life exposures including the feeding of a maternal diet of restricted protein content [4], or deficient in iron [5], sodium [6], zinc (Hanna et al, 2010), calcium [7]; high in saturated fat [8] and global nutrient restriction [9]. A number of mechanisms to explain nutritional programming of organ function have been proposed, including remodelling of tissues to reduce functional capacity [10], [11], [12], [13] and epigenetic modification of the genome [14], [15]. However, the majority of studies characterise *postnatal* gene expression or tissue function, potentially only capturing downstream phenotypes which may be secondary phenomena. Importantly, despite the diversity of these nutritional insults and the rodent strains used, the phenotypic outcomes in the offspring are remarkably similar, with the majority of models demonstrating a reduced nephron number and elevated systolic blood pressure (Langley-Evans & McMullen, 2010). The observed commonality of phenotype between models following very diverse nutritional or hormonal insults suggests that a relatively small number of common changes are occurring in response to nutrient availability *in utero*, to influence fetal development and determine long-term disease risk. We therefore propose that a small number of gatekeeper processes during development may underlie the nutritional programming observed in response to a range of insults.

In contrast to work which has used a candidate gene approach to try to explain the association between fetal nutrition and later physiological function and disease risk, we opted for a powerful, unbiased strategy to identify the fundamental biological responses to nutritional insult. The aim of the study was to systematically seek out common embryonic ‘gatekeeper’ genes and proteins for which expression changed uni-directionally in response to both protein and iron deficiency during a critical period of pregnancy. Most importantly we aimed to pinpoint the pathways and processes to which these gatekeepers contributed. Both dietary insults are associated with a common phenotype of raised blood pressure [3], [16]. The findings were further refined by looking for changes common to two strains of rat using a cross-over design incorporating two established programming models: protein deficiency in Wistar rats [4] and iron deficiency in Rowett Hooded Lister (RHL) rats [5]. We hypothesised that a small number of pathways would have altered expression in response to both dietary insults and in both strains of rat, and that these pathways would indicate putative gatekeeper processes. As males have been shown in many experiments to be more vulnerable to developmental programming effects [17], measurements were collected in male offspring only. The first section of the study aimed to confirm the long-term programming effects of prenatal iron and protein restriction, and establish whether this phenotype was consistent in response to both insults across both strains. Given the reported co-association of raised blood pressure with reduction of nephron number following maternal undernutrition [18], the latter was selected as a marker of the programmed phenotype. The second section of the study focused on embryonic tissues, with the aim of identifying gene or protein expression changes which occurred in all those groups which had demonstrated the adult phenotype. Our intention was to use a systematic approach to identify the common processes that respond to diverse nutritional interventions in embryonic life, and which may therefore drive the programmed outcome. It was considered possible that the key changes in expression of genes, proteins and pathways at the time of insult could be transient and impact only on early tissue development. By analysing gene and protein expression at the time of nutritional intervention, this study therefore had the potential to identify the initial drivers of long-term programming rather than characterising secondary events. However, whilst lasting expression changes in adult tissue were not necessarily expected, the study also considered the potential for undernutrition to impact on key processes in the longer-term.

## Materials and Methods

### Ethical Approval

All animal experiments were performed in the BioResources Unit of the University of Nottingham, under license from the United Kingdom Home Office in accordance with the 1986 Animals (Scientific Procedures) Act. The study was approved by the UK Home Office (Project Licence PPL40/2990) and University of Nottingham Ethics Committee (approval ID SLE/005/07).

### Animals

Female virgin Wistar rats (Harlan, UK) and Rowett Hooded Lister (RHL) rats (Rowett Institute of Nutrition and Health, UK) were subjected to a 12 h light (08:00–20:00)-dark (20:00–08:00) cycle at a temperature of 20–22°C with *ad libitum* access to food and water. For 4 weeks prior to mating, half of each strain of rat was fed either a control iron (50 mg Fe/kg; FeC) diet or an iron deficient diet (7.5 mg Fe/kg; FeD; [5]) to ensure depletion of iron stores during pregnancy. At a weight of approximately 180–200 g, females were mated with stud males of the same strain. After conception, determined by the presence of a semen plug on the cage floor, females were single-housed and 16 animals per strain were fed one of four diets as shown in Table 7: a control 18% (w/w) casein protein diet (CP), a 9% (w/w) casein (MLP) diet, or continuation of the iron diets described above, throughout gestation, as described previously [11]. Protein and iron-restriction diets were isocaloric relative to their specific controls, which differed by source of fat and type of carbohydrate. During pregnancy the animals were weighed and food intake was recorded daily.

### Section 1: Characterisation of Long-term Effect of Maternal Protein/Iron restriction

From day 13 of pregnancy, the animals on experimental diet (MLP and FeD groups, 8 dams/group) were switched to the corresponding control diet for the remainder of gestation. The control animals (CP and FeC, 8 dams/group) remained on the control diet. This meant that the period of undernutrition matched that for the second section of the study. At birth (day 21) all of these animals were transferred to the same standard laboratory chow diet (Teklad Global 2018, Harlan, UK). Litters were sexed, weighed and culled to eight pups (4 males and 4 females where possible) to ensure a standard level of nutrition during the suckling period. Tissues were harvested from culled pups. Offspring were weaned onto chow at 3 weeks of age and culled to one male and female per litter. Again, tissue was harvested from excess animals. Choice of which animals to cull was random. The remaining male offspring were culled for tissue collection at 16 weeks of age. Left kidneys were excised, weighed and fixed in 4% formaldehyde (Sigma). A section of kidney was cut, weighed and incubated in 1 M HCl for 30 minutes at 37°C before being homogenised in 5 ml 50 mM phosphate-buffered saline (pH 7.4). Glomeruli were counted in 3 replicates of cell suspension and total nephron number per kidney calculated as described previously [19].

#### Electron Microscopy of Kidney samples

To ascertain whether the effect of maternal diet on the kidney was isolated to impairment of nephrogenesis, glomerular ultrastructure was assessed by electron microscopy in a separate sample of Wistar offspring. At 14–15 weeks of age, male Wistar offspring of CP and MLP pregnancies (n = 5 per group) were culled and kidney tissue was placed in primary fixative (2% glutaraldehyde, 2% paraformaldehyde in 0.1 M sodium cacodylate buffer pH 7.35). Samples were processed for transmission electron microscopy and three glomeruli per animal were evaluated using images at 2550 and 16500 times magnification. The analysis of ultrastructure was adapted from the method of Whaley-Connell *et al.*, (2006) to assess the number of slit pores per 100 µm of glomerular basement membrane, slit pore diameter and foot process base width. Glomerular basement membrane thickness was assessed using the method of Neumann *et al.*, (2004).

### Section 2: Characterisation of Potential Gatekeeper Genes and Proteins

#### Tissue collection and preparation

On day 13 of gestation, 8 pregnant females per group were culled by CO_2_ asphyxia and cervical dislocation. Individual embryos and placentas were harvested. Tails were removed from embryos to establish sex. Tissues were snap frozen in liquid nitrogen and stored at −80°C. PCR was used to verify presence or absence of the sex determining region-Y (SRY) gene in lysed embryo tail tissue [20]. This gene is only expressed in males. All male embryos within a litter were pooled in order to obtain a better representation of maternal effects upon the whole litter. RNA and protein were extracted from the same embryo sample pool using the Protein and RNA Isolation System (PARIS, Ambion). Briefly, pooled embryos were homogenised in cell disruption buffer on ice. Half of each sample was retained as protein while half was used to isolate RNA using a spin column. Protein was desalted using spin columns (Pierce) and concentration determined by Lowry assay. All samples were diluted to 300 µg with the cell disruption buffer. RNA was DNase treated with Turbo DNase (Ambion) and integrity was checked on an agarose gel and a Nanodrop spectrophotometer (Thermo Fisher). All RNA samples were diluted to 100–500 ng/µl with RNase-free water (Sigma).

#### Microarray

An Affymetrix Genetitan Rat 230 microarray was performed by Service XS. This array comprises over 31,000 probe sets, analysing over 30,000 transcripts and variants from over 28,000 well-substantiated rat genes, as well as positive and negative controls. Before the labelling process, the integrity of all RNA samples was further checked using the Agilent 2100 Bioanalyser. Output data were supplied as Affymetrix CEL files and loaded into Genespring (Agilent). Data were normalised to QC controls and samples assigned to the appropriate diet/strain group. Targets were classified as a putative gatekeeper only if significant changes in gene expression (P<0.05, analysed by ANOVA) between a treatment (FeD or MLP) and its corresponding control (FeC or CP) were observed in the same direction (i.e. up-regulated or down-regulated) in response to both protein and iron deficiency or within both strains of rat (i.e. low protein or iron deficiency had the same effect in both strains). Data were loaded into MetaCore (GeneGo) to identify common pathways affected by both protein and iron deficiency or within both strains. The pathway analysis was also used to identify key transcription factors which formed ‘hubs’ in these common pathways. A list was constructed of genes associated with these hubs and this was cross-matched to the list of genes identified as putative gatekeepers from the microarray. A total of 36 genes appeared on both lists, and these were considered the most important gene targets for follow-up work. All microarray data is MIAME compliant and the raw data has been deposited in ArrayExpress (accession number E-MTAB-664).

#### Real-time PCR

Real-time PCR was performed on the same embryonic RNA sample that was prepared for microarray, to confirm the gene expression changes observed in the microarray analysis, focusing on the 36 gene targets identified above. In addition, 4 transcription factors which formed important “hubs” in the pathway analysis were selected as targets. Real-time PCR measurements were repeated in kidney tissues collected at birth and at 3 and 16 weeks of age to determine whether gene expression changes observed at the time of dietary restriction were maintained in the longer term. Seven genes and 4 transcription factors were selected for analysis at these postnatal time-points based on their expression in embryonic tissue (greater than 150% change between control and experimental diet) or importance in gatekeeper processes. RNA was reverse transcribed using Moloney murine leukemia virus (MMLV) reverse transcriptase (Promega) and real time PCR was performed using Probe Master Mix (Roche) and Taqman Rat Custom Expression Assays (ABI) on a Lightcycler 480 (Roche) in 384-well optical reaction plates. Expression values and linearity were determined using a cDNA standard curve. Data were normalised to total cDNA levels measured by Oligreen reagent (Invitrogen) at 80°C [21].

#### Cell cycle array

As the gene targets identified from the microarray and pathway analysis suggested that regulation of the cell cycle was a key gatekeeper process in embryos of the RHL strain, a pathway focussed RT^2^ Profiler PCR Array for Rat Cell Cycle (SABiosciences) was performed as further validation in these four groups. 500 ng of previously prepared embryonic RNA from RHL groups (CP, MLP, FeC and FeD; n = 6 for each) was reverse transcribed using a RT^2^ First Strand kit (SABiosciences). Real time PCR was performed on cDNA in SYBR green mix in customised 384-well array plates to analyse expression of 84 genes key to cell cycle regulation and housekeeping genes.

#### Proteomics

Proteomic analysis was carried out by the Proteomics Section at Rowett Institute of Nutrition and Health. Protein samples were loaded onto sixty- four 8–16% acrylamide gels to separate proteins in each embryo sample by isoelectric focussing in the first dimension (pI range 3–10) and SDS-PAGE in the second dimension. Gels were stained with the Colloidal Coommasie stain method, and imaged on a Bio-Rad GS800 scanning densitometer, followed by analysis using Progenesis SameSpots software. Each image was quality assessed before selection of a ‘master gel’, to which the other 63 gels were aligned. Gels were assigned to one of the 8 experimental groups and any spots with differences in area and density between groups were identified by ANOVA. Again, spots were only classed as putative gatekeeper proteins when they were similarly differentially up- or down-regulated with both dietary insults or across both strains. Spots of interest were hand-cut from the SDS-PAGE gels. In-gel digestion and trypsinisation of the cut spots was performed on a Proteome Works System, Mass PREP Station Robotic Handling System and extracted peptides were analysed on a nano LC-MS/MS system using Q-Trap. The total ion current data was searched against the MSDB database using the MASCOT search engine (Matrix Science) with the following search criteria: allowance of 0 or 1 missed cleavages; peptide mass tolerance of ±1.5 Da; fragment mass tolerance of ±1.5 Da, trypsin as digestion enzyme; carbamidomethyl fixed modification of cysteine; methionine oxidation as a variable modification; and charged state as 2^+^ and 3^+^. The top MASCOT results were further selected for best matches with criteria of protein identifications having a Mascot score higher than 40 (threshold) and more than one peptide match. The identification was considered only with a combination of the highest Mascot score and maximum peptide coverage.

#### Statistical Analysis

Total nephron counts, gene expression changes and protein spot area and density were compared across diet/strain groups by one-way ANOVA in SPSS v16.0. In the event of a significant ANOVA outcome (P<0.05), Bonferonni post-hoc tests were performed.

## Results

### Section 1: Characterisation of Long-term Effect of Maternal Protein/Iron restriction

Litter size averaged 10 pups per dam, and there were no significant differences in reproductive outcomes between the 8 groups (data not shown). Average birth weight of male pups was greater for RHL neonates than Wistars (P<0.03), but again there was no difference between dietary groups within each strain (Table 1). By 16 weeks of age, body weight was significantly greater in males which had been exposed to FeC or FeD during pregnancy compared to those exposed to CP or MLP (P<0.01). Kidney weight at this age did not differ between groups, even when adjusted for body weight.

**Table 1:** Male birth weight, 16 week body weight, 16 week left kidney weight, and kidney∶body weight ratio.

Group	W CP	W MLP	W FeC	W FeD	RHL CP	RHL MLP	RHL FeC	RHL FeD
n	8	7	8	7	8	6	7	6
Litter size	12.1±1.6	12.4±0.8	11.1±1.4	10.0±1.8	7.1±1.6	9.6±1.6	10.7±1.5	8.7±1.4
Birth weight (g)	4.9±0.2^a^	5.3±0.4	4.8±0.3^a^	4.8±0.2^a^	6.4±0.4^b^	6.3±0.2	6.2±0.3	6.6±0.2^b^
16 week body weight (g)	386±12^a^	397±9^a^	502±11^b^	493±16^b^	422±21^a^	452±11^a^	526±17^b^	549±18^b^
16 week left kidney weight (g)	1.2±0.1	1.4±0.2	2.1±0.5	1.5±0.1	1.4±0.1	1.2±0.01	1.6±0.1	1.7±0.1
Kidney∶Body weight (mg)	3±1	4±1	4±1	3±1	3±1	3±1	3±0.3	3±1

**Data expressed as mean ± SE, a<b P<0.05.**

**Figure 1:**
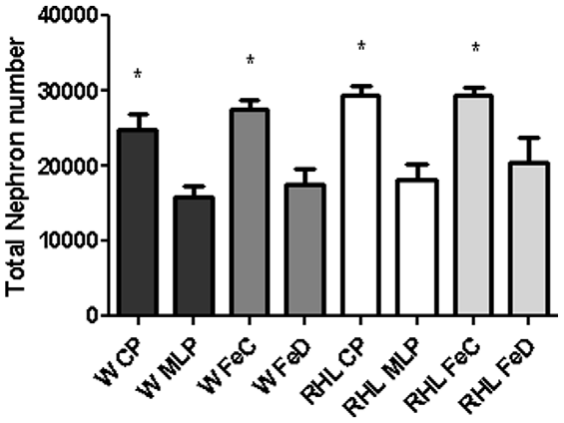
Nephron numbers in each diet/strain group. Data are mean ± SE. *P<0.05 between control and corresponding deficient diet. n = 8 animals per group.

In both strains of rat, prenatal protein (Wistar: P<0.005; RHL: P<0.001) or iron (Wistar: P<0.005; RHL: P<0.05) restriction led to a significant reduction in nephron endowment at 16 weeks of age, compared to exposure to the equivalent control diet (Figure 1). This confirmed a phenotypic marker in common to both dietary restrictions in both strains of rat. Qualitative assessment of podocyte structure in TEM images of kidneys from Wister rats at 2550 times magnification revealed no gross evidence of injury such as vacuolation or the presence of lysosomes and autophagosomes. As shown in Table S1, exposure to a maternal low protein diet had no significant effect on glomerular basement membrane thickness, foot process base width, slit pore diameter or the numbers of slit pores. As such, the effect of protein restriction on the kidney appeared to be isolated to impairment of nephrogenesis, with no major impact on glomerular ultrastructure by 16 weeks of age.

### Section 2: Identification of Putative Gatekeepers

#### Microarray

The number of embryos collected from each dam averaged 10. There was no difference in the number of embryos per litter across the 8 strain/diet groups. The microarray did not identify any genes which changed significantly in the same direction with both dietary insults and in both strains. However, expression of 2 genes significantly changed in the same direction for both prenatal protein and iron restriction in Wistar rats and 67 in RHL rats. Seventeen genes changed uni-directionally with protein restriction in both strains of rat, and 68 with iron restriction in both strains (Table 2). Fold-changes were small, ranging from 10 to 90% changes up or down, when assessing both diets within a strain or the same diet across both strains, or to 180% up-regulation when considering the effect of one diet manipulation within one strain of rat. As no changes occurred in common to both diets and strains, strain (both diets) or diet (within both strains) specific gatekeepers were identified. A full list of putative gatekeeper genes identified at this stage is provided in Table S2a–d.

**Table 2:** Number of genes up- and down-regulated with each dietary insult compared to its control, in each strain of rat (n = 8 animals per group; RHL – Rowett hooded Lister, MLP – maternal low protein, FeD – iron deficient).

	*Wistar*			*RHL*			*Both Strains*		
	Genes Up-regulated	Genes Down-regulated	Total	Genes Up-regulated	Genes Down-regulated	Total	*Genes Up-regulated*	*Genes Down-regulated*	*Total*
MLP	431	291	722	951	1256	2207	*6*	*10*	*16*
FeD	1082	1013	2095	1437	815	2252	*47*	*21*	*68*
**Both Diets**	*1*	*1*	*2*	*40*	*27*	*67*	*0*	*0*	*0*

Italic boxes highlight numbers of potential gatekeeper genes.

#### Pathway analysis

Pathway analysis was performed using the MetaCore platform to identify the functional processes that were perturbed by exposure of the embryo to maternal undernutrition. This analysis was based solely upon the putative gatekeeper genes identified from the microarrays (Table S2a–d). There were insufficient Wistar specific gatekeeper genes to find any pathways common to prenatal protein and iron restriction in that strain. For RHLs, however, the most significant pathways were concerned with cytoskeleton remodelling, cell cycle processes, apoptosis, signal transduction, glycogen metabolism and developmental processes (Figure S1a). Both strains of rat exposed to prenatal protein restriction shared pathways concerned with slit-robo signalling, clathrin coated vesicle formation, cell adhesion, and again cytoskeleton remodelling (Figure S1b). For iron deficiency, both strains of rat had common pathway changes in line with those reported for RHLs and protein restriction (clathrin coated vesicle formation, cytoskeleton remodelling, cell adhesion and developmental processes), in addition to endosome formation, transcription, muscle contraction and immune response (Figure S1c).

The pathway analysis also identified transcription factors which formed ‘hubs’ in the common pathways. A list was constructed of genes associated with these hubs and this was cross-matched to the list of genes identified as putative gatekeepers from the microarray. A total of 36 genes appeared on both lists, and these were considered the most important gene targets for follow-up work. In addition, 4 transcription factors which formed important “hubs” in the pathway analysis were selected as targets.

#### Real-time PCR

Real-time PCR analysis of the 36 gene targets and 4 transcription factors identified in the microarray and pathway analysis generally failed to demonstrate the statistical significance of the microarray results, although the changes did show general agreement (Tables 3 and 4). Of the thirty-six genes selected, sixteen were shown by PCR to be up- or down-regulated with the same direction of change indicated by the microarray, across both dietary insults within a strain or the same insult in both strains. Of these, one gene, *Ube2c*, demonstrated significant down-regulation (approximately 50%) for both iron and protein restriction in embryos from RHL pregnancies. A further four of these sixteen genes (*Acvr2b*, *Nufip1*, *Rps20* and *Taf13*) exhibited significant changes between one of the two pairs in the group. For example, *Nufip1* was significantly down-regulated in MLP vs CP in RHLs (P<0.004) but there was no significant difference for FeD vs FeC (P<0.3) (i.e. partial verification of the gatekeeper categorisation based upon the microarray).

**Table 3:** Expression of selection of microarray genes in embryonic tissue analysed by real-time PCR in response to iron deficiency in both Wistar and Rowett Hooded Lister rats (FC = fold-change, RHL = Rowett Hooded Lister).

Gene symbol	Wistar		RHL	
	P	FC	P	FC
*Acta1*	0.38	1.48	0.15	0.78
*DBI*	0.24	1.18	0.33	0.73
*Disp1*	0.29	1.02	0.34	0.74
*Eef1g*	0.11	0.73	0.14	2.50
*Eef2b1*	0.12	0.69	0.84	0.94
*Esm1*	0.34	1.08	0.15	0.65
*GMPS*	0.33	1.27	**0.09**	0.59
*Hint1*	0.14	0.56	0.14	4.00
*Mxi1*	0.93	1.02	0.96	0.89
*Nol7*	**0.03**	0.62	0.79	1.17
*Opa1*	0.45	1.16	0.40	0.75
*Prmt2*	0.80	1.24	0.59	1.21
*Rnf7*	**0.08**	0.33	0.15	2.34
*Rps20*	**0.04**	0.92	0.90	0.97
*SDCCAG10*	0.26	1.10	0.16	0.66
*SMPD3*	0.38	1.10	0.87	1.06
*Stx12*	0.7	0.86	0.10	2.88
*Taf13*	**0.01**	0.66	0.38	0.79
*Tomm20*	0.93	1.03	0.44	1.27
*USMG5*	0.66	1.24	**0.01**	0.61
*XBP1*	0.65	1.46	0.76	1.10

The selected genes had all been shown to be significantly up- or down-regulated by whole genome microarray. n = 8 animals per group.

**Table 4:** Expression of selection of microarray genes in embryonic tissue analysed by real-time PCR in response to iron deficiency in Rowett Hooded Lister rats (FC = fold-change).

Gene symbol	Protein		Iron	
	P	FC	P	FC
*Acvr2b*	**0.02**	3.35	0.28	1.65
*C-Myc*	0.35	0.64	**0.08**	0.66
*Ccnh*	0.26	1.77	0.44	1.36
*Eif2b1*	0.52	0.65	0.58	0.85
*HNF4a*	**0.07**	0.79	0.42	0.66
*Mcart1*	0.34	0.74	0.82	0.93
*Nufip1*	**0.00**	0.53	0.30	0.54
*p53*	0.30	2.82	**0.08**	5.42
*PMEPA1*	0.55	0.87	0.69	1.15
*Pole3*	0.41	0.80	0.38	0.57
*Prmt3*	0.85	0.70	0.58	0.72
*Psat1*	0.22	0.71	0.10	0.60
*Securin*	0.52	1.30	0.48	0.64
*SP1*	0.74	0.71	**0.06**	0.51
*SUCLG1*	0.87	0.75	0.40	0.79
*TBX3*	0.47	0.86	0.15	0.75
*Tomm34*	0.28	0.66	0.11	0.69
*Txndc12*	0.35	0.82	0.77	0.83
*Ube2c*	**0.02**	0.55	**0.03**	0.45

The selected genes had all been shown to be significantly up- or down-regulated by whole genome microarray. n = 8 animals per group.

Sixteen of the thirty-six genes agreed with the microarray for one of the pairs within the group, two significantly so, but showed the opposite direction of change in the other pair (again, a partial verification of the microarray study). Only four of the thirty-six genes exhibited changes in expression that were in the opposite direction to the microarray (*Cyclin H*, *Mcart1*, *TBX3* and *Tomm20*). Four genes (*Eef1g*, *Hint1*, *Rnf7* and *Stx12*) were suggested by the real-time PCR analysis to be gatekeepers for a different group than that originally identified by microarray (RHL gatekeepers rather than iron restriction gatekeepers). Two of these (*Hint1* and *Rnf7*) now showed significant changes between RHL CP and MLP. The four transcription factors which were selected from the pathway analysis and measured by real-time PCR (*SP1*, *C-Myc*, *HNF4a* and *p53*) all showed uni-directional changes in expression with both dietary insults in RHL rats, and changes in expression approached statistical significance for at least one of the dietary insults in each case (P<0.06 to 0.08).

On the basis of the combined microarray and real-time PCR analyses we were able to identify twenty genes and four transcription factors which were likely gatekeeper genes for either iron restriction (*Eef2b1*, *Prmt2*, *Rps20*, *SMPD3*, *Taf13*, *Xbp1*), and for both iron and protein restriction in RHL rats (*Acvr2b*, *C-myc*, *Eef1g*, *Eif2b1*, *Hint1*, *HNF4a*, *Nufip1*, *p53*, *Pole3*, *Prmt3*, *Psat1*, *Rnf7*, *Sp1*, *Stx12*, *Suclg1*, *Tomm34*, *Txndc12* and *Ube2c*).

Real-time PCR measurements were repeated in kidney tissues collected at birth and at 3 and 16 weeks of age to determine whether gene expression changes observed at the time of dietary restriction were maintained in the longer term. The four transcription factors were all followed up to assess their importance as “hubs”. *Hint1*, *eef1g*, *Acvr2b*, and *stx12* were selected for this analysis as they demonstrated greater than 150% changes in expression in embryo tissue. *pttg1* and *Cyclin H* were chosen to represent the cell cycle processes, specifically sister chromatid cohesion and initiation of mitosis, which were identified by pathway analysis. *Tbx3* was followed up due to its roles in the degradation of *p53* and cell cycle arrest. In neonates exposed to a low protein diet *in utero*, it was generally the case that differential expression of the transcription factors occurred in the opposite direction to the changes seen in embryos (i.e. up-regulated genes became down-regulated and vice versa). In neonates exposed to iron restriction, patterns of transcription factor expression were similar to those observed in the embryos, but the target genes tended to show changes in direction of expression compared to the embryonic period. This pattern persisted through to three weeks of age. By 16 weeks of age, there were few differences in expression that were consistent with the embryonic effects (Table S3).

As the gene targets identified from the microarray and pathway analysis suggested that regulation of the cell cycle was a key gatekeeper process in embryos of the RHL strain, a pathway focussed rat cell cycle array was used to verify if the expression of wider components of this system are altered in early life programming. RHL embryos were selected for this analysis as there was more robust evidence of the cell cycle being a gatekeeper process in this strain. The targeted array included 84 cell cycle-associated genes. Analysis of the array results identified a number of significant changes in expression, most of which were down-regulation of genes in embryos exposed to maternal protein restriction. The functions of nine differentially expressed genes were spread across the G1 phase and G1/S transition (*Taf10*), the S phase (*Rad17*), the M phase (*ccnb1*, *Ran*, *Wee1*) and the cell cycle checkpoint and cell cycle arrest (*Apbb1*, *Mdm2*, *Pmp22*, *Tsg101*). *Cyclin B1* was significantly down-regulated by protein restriction, and there was also a tendency towards down-regulation (P = 0.077) in iron deficient embryos. This target was of particular interest as the initial Affymetrix microarray analysis had also identified it as an iron-deficiency gatekeeper gene. *Pmp1d* and *Casp3* were significantly up-regulated in FeD tissue compared to FeC. In addition to the nine genes for which significant changes in expression were noted a further five, including *ccna2* and *cdk4*, were found to be down-regulated to the same degree in MLP embryos, but without achieving statistical significance (P<0.058 to 0.09). Genes of interest are shown in Table 5 and data for all genes in the array are shown in Table S4. The pathway-focused array therefore provided evidence that the regulation of the cell cycle is a significant target for effects of both iron deficiency and protein restriction. Coupled to the data from the whole genome array and the real-time PCR, this focused analysis is strongly suggestive that events preceding mitosis are influenced by maternal undernutrition. Although *Ube2c* was not included in the cell cycle array, this gatekeeper gene identified in the earlier analyses plays a key role in regulating cell cycle processes, driving elimination of cyclins via ubiquitinylation and proteasome action.

**Table 5:** Selection of gene expression data from RT^2^ Profiler Rat Cell Cycle PCR Array (FC = fold-change, RHL = Rowett Hooded Lister).

Gene	RHL Protein		RHL Iron	
	p-value	FC	p-value	FC
Apbb1	**0.01**	0.5	0.76	1.07
Casp3	0.19	0.65	**0.05**	1.20
Ccna2	0.08	0.56	0.18	1.16
Ccnb1	**0.04**	0.56	0.08	0.78
cdk4	0.09	0.62	0.30	1.11
Gadd45a	0.07	0.61	0.41	0.92
Mdm2	**0.04**	0.64	0.25	1.09
Nek2	0.09	0.61	0.87	1.03
Pmp22	**0.03**	0.56	0.97	1.01
Ppm1d	0.20	0.68	**0.03**	1.24
Rad17	**0.05**	0.65	0.81	1.03
Rad9	0.06	0.64	0.92	1.02
Ran	**0.03**	0.63	0.80	1.09
Taf10	**0.01**	0.60	0.68	0.97
Tsg101	**0.04**	0.60	0.22	1.12
Wee1	**0.04**	0.56	0.96	0.99

All data is shown in Table S3. n = 6 animals per group.

#### Proteomics

Similarly to the microarray analysis, the proteomics analysis did not identify any proteins differentially expressed across both strains and diets. However, 9 proteins were expressed at significantly different levels with both diets in Wistar rats and 8 in RHL rats (Table 6). Some of the proteins identified were related to the same processes that had emerged from the gene array analyses. Most notably these included cytoskeletal functions (actin-related protein 3 and tubulin α-1 chain) and protein degradation via proteasome 26 (SUG1 and 26S proteasome β type 1). The latter was a process common to both protein and iron restriction in both rat strains.

**Table 6:** Proteins identified by mass spectrometry following 2D gel electrophoresis.

Strain	Direction	Gatekeeper Protein common to low protein and iron deficient diets	Function
Wistar	Up	Protein disulfide isomerase ER60 precursorThioredoxin-like 2ARP3 actin-related protein 3 homologGuanine nucleotide-binding proteinG(i) alpha-1, chain AAlpha-fetoproteinTubulin alpha-1 chain	Protein foldingMembrane trafficking – cytoskeletonEmbryogenesis, growth regulationCytoskeletal protein
Wistar	Down	Peptidylprolyl isomerase CSUG1 protein	Protein folding26S Proteasome subunit – peptide degradation
RHL	Up	LonPhosphoserine aminotransferase26S Proteasome subunit, beta type 1	Protein degradationAmino acid biosynthesisPeptide degradation
RHL	Down	Methionine aminopeptidase 2 (Initiation factor 2-associated 67 kDa glycoprotein) (p67) (p67eIF2)Dihydrolipoamide S-acetyltransferase (E2 component of pyruvate dehydrogenase complex)Farnesyl-pyrophosphate synthetase, testisPyruvate dehydrogenase (Lipoamide) beta1MABA f1-atpase alpha chain alpha chain A	Regulator of translationIsoprenoid biosynthesisEnergy metabolismEnergy metabolism

**These proteins were significantly up- or down-regulated following prenatal protein and iron restriction within each strain of rat (RHL – Rowett Hooded Lister). n = 8 animals per group.**

**Table 7:** Feeding regime of pregnant dams across 8 experimental groups.

*Group*	*n*	*Strain*	*Maternal Diet D0-13*	Maternal Diet D13-birth (where applicable)
W CP	16	Wistar	CP	CP
W MLP	16	Wistar	MLP	CP
W FeC	16	Wistar	FeC	FeC
W FeD	16	Wistar	FeD	FeC
RHL CP	16	RHL	CP	CP
RHL MLP	16	RHL	MLP	CP
RHL FeC	16	RHL	FeC	FeC
RHL FeD	16	RHL	FeD	FeC

8 dams/group were culled at day 13 of pregnancy (characterisation of potential gatekeepers), 8 dams/group proceeded until term (characterisation of long-term effects). (RHL - Rowett Hodded Lister; CP – control protein; MLP – maternal low protein; FeC – control iron; FeD – low iron.)

## Discussion

As far as the authors are aware, this is the first study to use a multi-strain and multi-diet cross-over approach to identify common molecular mechanisms by which differing maternal nutrient restrictions can exert similar long-term nutritional programming effects. A critical period was selected for the nutritional insults, which corresponded to the development of key renal and cardiovascular processes such as formation of the metanephros, blood pressure control and extensive development of the vascular and renal systems [22], [23], [24]. Unlike many other studies in the field, genomic and proteomic changes were captured in the embryo, at the actual time of the insult and initial programming stimuli, rather than later in life when secondary effects can mask the primary mechanisms. The aim of the study was to identify gatekeeper genes and proteins and the associated pathways and processes that may drive the programming response to nutritional insult. The novel outcome of the work was a diverse raft of evidence to support the idea that regulation of the cell cycle, cytoskeletal remodelling and protein degradation are key targets for programming insults.

The first section of the study showed that feeding pregnant rats a diet deficient in either protein or iron until mid-gestation resulted in an impaired nephron endowment in their male adult offspring, in both strains studied. Thus we demonstrated a common programmed phenotype, which is consistent with our previous reports that both dietary insults induce hypertension in rats [4], [5]. This occurred in the absence of ultrastructural changes in offspring of protein restricted rats and we infer that undernutrition impacts upon nephrogenesis (i.e. early events), rather than through injury to existing structures that occurs in response to secondary phenotypic features such as raised blood pressure [25].

The main body of the study involved searching for gatekeeper genes, proteins and pathways which were significantly up- or down-regulated in a nutrient-deficient group compared to its corresponding control, in the same direction for both dietary manipulations and in both strains. The intention was not to identify biomarkers of protein or iron deficiency, but to examine whether fundamental developmental processes were responsive to diverse nutritional stressors. As no changes occurred in common to both diets and strains, strain (both diets) or diet (within both strains) specific gatekeepers were identified. These assessments were made in embryos at a time corresponding to early stages of nephrogenesis. While the overall numbers of gene changes within each pairwise comparison were vast, a smaller number of gene expression changes were common to both diets, or both strains. Coupled to the equivalent protein expression changes and similarity of the identified pathways, this gave us a powerful experimental approach with which to home in on certain processes which were altered in common with both dietary insults and with two separate strains of rat. Although the gatekeeper genes and proteins identified were all either strain or diet specific, the power of the experimental design, assessing gene and protein expression in the same samples, meant that the overview showed consistency in the processes impacted by undernutrition. Thus, although the specific components of the cell cycle regulatory pathway that were differentially expressed in response to protein restriction were not the same as those differentially expressed in response to iron deficiency, we can state with confidence that the cell cycle and hence cell proliferation within developing tissues are a programming target.

As expected, most of the changes in expression which were identified in the embryo did not appear to persist into postnatal life. The changes observed in the whole embryo, a heterogeneous mixture of different cell types, many of which not terminally differentiated, may have been specific to non-renal tissue, whilst the postnatal measurements were made using a single organ, albeit with diverse cell types. Alternatively, it may be that transient changes in gene expression during critical developmental periods are important, as they may result in widespread tissue remodelling akin to the impaired nephrogenesis we observed. It is important for further work to be carried out to assess the localization of the gene and protein changes within embryos. It was decided to utilise entire embryos for our analyses, despite the high proportion of the embryonic body which constitutes developing brain tissue. It is interesting to note the absence of brain-specific targets within the genes identified as putative gatekeepers. In terms of fetal growth, a brain-sparing effect has been observed in various models of pregnancy undernutrition [26], whereby brain growth is preserved at the expense of truncal organs. It is possible that this occurs because brain-specific genes are relatively insensitive to manipulation of nutrient supply.

The most significant process observed to be perturbed by exposure to both diets in RHL rats, and across both strains with either iron deficiency or protein deficiency, was cytoskeletal remodelling. The two main components of the cytoskeleton are actin and tubulin, which were identified as differentially expressed gatekeeper targets by microarray and proteomics respectively, along with the actin-related protein (Arp) 2 and 3 complex. Cytoskeletal dynamics are partially controlled by Slit-Robo signalling via regulation of the Rho family GTPases, both identified as significant pathways in our analyses. Mice lacking genes for either *Slit* or *Robo* suffer fatal kidney defects soon after birth [27]. Given the other findings of the pathway analysis and the cell cycle focused array, the association of the cytoskeleton with cell cycle regulation is perhaps of greater significance. Correct integrity of the cytoskeleton and the Arp 2/3 complex are necessary to ensure correct assembly of the mitotic spindle and allow progression through the G2 checkpoint of the cell cycle and initiate mitosis [28].

Regulation of the cell cycle was identified as a significant gatekeeper pathway, in embryos exposed to both prenatal protein and iron restriction. During mitosis, clathrin-coated vesicle formation (another significant gatekeeper process) is shut down and clathrin is instead utilised to stabilise the mitotic spindle [29] by binding with tubulin. Clathrin aids the congression of chromosomes during sister chromatid cohesion, another significant pathway identified in our analysis. The degradation of *securin* and *cyclin B1*, (identified as a gatekeeper gene in both microarray and PCR array analysis), is then required for the separation of sister chromatids and the progression of the cell cycle. This process is carried out by the proteasome 26 complex, components of which were identified as putative gatekeepers by proteomic analysis. The proteasome complex is associated with the actin cytoskeleton [30], degrading proteins which are damaged or no longer required. Proteins are tagged for degradation by ubiquitin and the process catalysed by ubiquitin-conjugating enzymes such as *Ube2c*, which was shown to be down-regulated by both protein and iron-deficiency in RHLs in our microarray and by real-time PCR. The proteasome 26 complex determines how long transcription factors remain active. A number of transcription factors, such as *C-myc*, *SP1* and *HNF4α* were identified as important hubs in the significant gatekeeper processes, so their longevity could be an important factor in the regulation of these processes. These transcription factors were all down-regulated in MLP and FeD tissue compared to the controls when measured by PCR.

Several genes (*Xbp1*, *Tomm34*, *NRF-1*) which were differentially regulated by maternal diet play an important role in cell cycle processes. Cell cycle control and maintenance of DNA integrity were also shown to be perturbed following protein restriction *in utero* as measured by microarray in neonate liver tissue (Clark, Langley-Evans, Bogdarina and Altobelli, unpublished observation). For these reasons, a pathway-specific cell cycle array was carried out on the embryo samples, to assess the general impact of undernutrition on cell cycle regulation in an unbiased manner. The PCR array highlighted that the cell cycle was particularly impacted by protein restriction. Specifically it appeared to be the mitotic checkpoints which were most vulnerable to the effects of undernutrition. The first checkpoint is located at the end of the G1 phase, restricting entry into the S phase if environmental conditions make cell division impossible. *Pmp22* and *Apbb1*, significantly down-regulated in MLP embryos in the PCR array analyses, are normally responsible for negatively regulating the S phase by inducing apoptosis. Down-regulation of these genes and others at the G1 checkpoint may allow damaged DNA to progress through the cycle.

The second checkpoint is located at the end of the G2 phase, triggering the initiation of mitosis. *Wee1* forms an important component of this checkpoint, inhibiting *cdk1* which, when bound to its cyclin partners, is crucial for entry into mitosis. Down-regulation of *Wee1*, as found in RHL MLP embryos by PCR array, may therefore allow mitotic progression while DNA replication or repair is incomplete. *Wee1* has an additional role in the cell size checkpoint, coordinating cell size and cell cycle progression. Loss of *Wee1* function could lead to premature cell division and smaller than normal daughter cells. *Rad17* and *Rad9*, down-regulated by protein deficiency, are also required at the G2/M checkpoint for DNA damage repair to restore the integrity of DNA prior to DNA synthesis or separation of the replicated chromosomes. The mitotic spindle checkpoint ensures that all chromosomes have aligned correctly and are properly attached at the spindle. *Ran* is responsible for directing spindle assembly to the correct chromosome position, and was significantly down-regulated in MLP embryos in the PCR array. *Tsg101*, also down-regulated in MLP embryos, colocalises with microtubule organising centres and mitotic spindles during mitosis. Deficiency of this gene has been associated with reversible abnormalities in microtubules, mitotic spindles and nuclei [31].

Two important cell cycle genes, *Ppmd1* and *Casp3*, were significantly up-regulated in FeD tissue compared to its control. Expression of *Ppmd1* is induced in a *p53*-dependent manner in response to environmental stresses, contributing to growth inhibition and suppression of stress induced apoptosis. *Casp3* plays a central role in the execution phase of cell apoptosis, thereby negatively regulating the cell cycle. Therefore over-expression of these genes following nutrient deficiency could lead to inappropriate control of programmed cell death in the developing embryo. Indeed, findings at the whole embryo level suggest that cell proliferation could be impaired with a greater number of cells becoming arrested at the G2/M checkpoint and most likely a greater degree of apoptosis. Welham et al., [19] concluded that protein restriction in pregnancy was associated with increased apoptosis of mesenchymal cells during metanephrogenesis, ultimately leading to a reduced glomerular endowment in the rat. Thus our study may have identified the fundamental driver of the tissue remodelling that is proposed to occur in response to maternal undernutrition (Langley-Evans & McMullen, 2010), for which evidence is readily available in the kidneys [11], pancreas [13] and brain [10], [32] of the offspring of protein-restricted dams.

Follow-up of key gene targets was performed postnatally, specifically in kidney tissue as opposed to the whole genome. PCRs showed that expression was in a state of flux between embryonic and early postnatal periods. Transcription factors which had been compromised during embryonic development underwent compensatory over-expression or down-regulation throughout early growth and development. This highlights that studies trying to unravel the consequences of nutrient manipulation during fetal life at a postnatal time-point will face problems with the sensitivity of their timing and choice of tissue of interest. This is an important outcome of the study that should be recognised by others in the field.

While assessment of microarray targets by PCR did not always result in similar magnitude and significance of changes in expression, it should be noted investigators are only just beginning to address issues of what criteria constitute a satisfactory verification of a microarray result [33]. This is recognised as a particular problem in nutrition studies, where effects can be expected to be small. Both array and PCR-based techniques are considered to have their own advantages and disadvantages, and may be better used together as complementary studies rather than considering the PCR result as validation of the array [34]. This approach was adopted in the current experiment and a major strength of this study lies in careful power analyses which ensured optimal sample size and biological robustness.

This study is of major importance to the understanding of the basic biological mechanisms underlying developmental programming, due to its thorough and unique experimental design. Using a high sample number, it allowed a systematic assessment of whole embryo protein *and* gene expression changes that occur in common across two established models and, crucially, during a critical period of organ development in fetal life. A number of mechanisms are already well-characterised to explain how early life nutritional signals promote increased disease risk. While these studies are all important, they characterise processes which mediate the downstream pathology or metabolic consequence of programming, rather than the actual basis of the programmed response itself. We have identified nutritional influences upon fundamental processes which will impact on cell type and functional number within tissues as a central feature of early life programming. These findings can now open the door for the targeted approach to confirm the processes involved, and the development of new disease prevention and treatment strategies. Further work investigating the specific organs or tissues where gene and protein expression changes take place is warranted.

## Supporting Information

Figure S1 **Statistically significant Go pathway maps from GeneGo.** Processes are ranked based upon p-value. Bars represent inverse log of the p-value. **S1A:** Significant GeneGo pathways common to both prenatal protein and iron restriction in RHL rats. **S1B:** Significant GeneGo pathways common to prenatal protein restriction in both Wistar and RHL rats. **S1C:** Significant GeneGo pathways common to prenatal iron restriction in both Wistar and RHL rats.
(TIF)

Table S1 **Podocyte ultrastructure measured from electron microscopy images.** Data expressed as mean ± SEM. GBM- glomerular basement membrane.
(DOCX)

Table S2 **Summary of genes which changed statistically significantly and uni-directionally in response to either both diets or in both strains of rat.** **S2a:** Summary of genes which changed significantly and uni-directionally in response to protein deficiency in both Wistar and Rowett Hooded Lister rats, as analysed by microarray of embryonic tissue. Of 16 genes which satisfied these criteria, 11 were unidentified (not shown). (FC = fold-change, RHL = Rowett Hooded Lister). **S2b:** Summary of genes which changed significantly and uni-directionally in response to iron deficiency in both Wistar and Rowett Hooded Lister rats, as analysed by microarray of embryonic tissue. Of 68 genes which satisfied these criteria, 21 were unidentified (not shown). (FC = fold-change, RHL = Rowett Hooded Lister). **S2c:** Summary of genes which changed significantly and uni-directionally in Wistar rats in response to both protein and iron deficiency, as analysed by microarray of embryonic tissue (FC = fold-change). **S2d:** Summary of genes which changed significantly and uni-directionally in Rowett Hooded Lister rats in response to both protein and iron deficiency, as analysed by microarray of embryonic tissue. Of 67 genes which satisfied these criteria, 25 were unidentified (not shown). (FC = fold-change)
(DOCX)

Table S3 **Real-time PCR determination of gatekeeper target gene expression in postnatal kidney tissue.** (W P = Wistar Proteins, W Fe = Wistar Irons, RHL P = Rowett Hooded Lister Proteins, RHL Fe = Rowett Hooded Lister Irons; FC = fold-change.)
(DOCX)

Table S4 **Gene expression data for RT^2^ Profiler Rat Cell Cycle PCR Array.** (FC = fold-change, RHL = Rowett Hooded Lister.)
(DOCX)
