# Supplementary figures and images for: Cell Cycle Regulation and Cytoskeletal Remodelling Are Critical Processes in the Nutritional Programming of Embryonic Development
Source: PLoS One. 2011 Aug 17;6(8):e23189. doi: 10.1371/journal.pone.0023189 (PMC3157362; doi:10.1371/journal.pone.0023189)

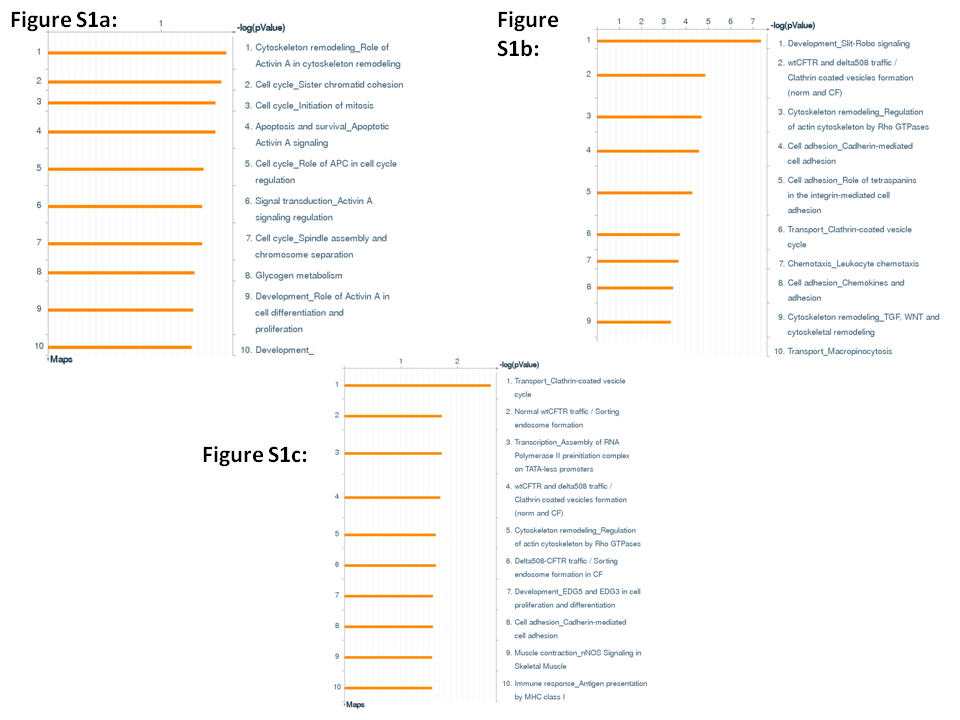

Supplement: Figure S1 — Statistically significant Go pathway maps from GeneGo. Processes are ranked based upon p-value. Bars represent inverse log of the p-value. S1A: Significant GeneGo pathways common to both prenatal protein and iron restriction in RHL rats. S1B: Significant GeneGo pathways common to prenatal protein restriction in both Wistar and RHL rats. S1C: Significant GeneGo pathways common to prenatal iron restriction in both Wistar and RHL rats. (TIF) [file pone.0023189.s001.tif]
