# Supplementary material for: Cell Cycle Regulation and Cytoskeletal Remodelling Are Critical Processes in the Nutritional Programming of Embryonic Development
Source: PLoS One. 2011 Aug 17;6(8):e23189. doi: 10.1371/journal.pone.0023189 (PMC3157362; doi:10.1371/journal.pone.0023189)
Supplement: Table S1 — Podocyte ultrastructure measured from electron microscopy images. Data expressed as mean ± SEM. GBM- glomerular basement membrane. (DOCX) [file pone.0023189.s002.docx]

**Table S1:**

|  | **Maternal dietary group** | |
| --- | --- | --- |
|  | **Control (n=4)** | **Low protein (n=5)** |
| GBM thickness (nm) | 203±18 | 191±14 |
| Slit pore diameter (nm) | 31.7±1.6 | 26.2±3.1 |
| Slit pores (pores per 100µm GBM) | 392±40 | 394±31 |
| Foot process base width (nm) | 294±39 | 257±26 |
