# Supplementary material for: Cell Cycle Regulation and Cytoskeletal Remodelling Are Critical Processes in the Nutritional Programming of Embryonic Development
Source: PLoS One. 2011 Aug 17;6(8):e23189. doi: 10.1371/journal.pone.0023189 (PMC3157362; doi:10.1371/journal.pone.0023189)
Supplement: Table S2 — Summary of genes which changed statistically significantly and uni-directionally in response to either both diets or in both strains of rat. S2a: Summary of genes which changed significantly and uni-directionally in response to protein deficiency in both Wistar and Rowett Hooded Lister rats, as analysed by microarray of embryonic tissue. Of 16 genes which satisfied these criteria, 11 were unidentified (not shown). (FC = fold-change, RHL = Rowett Hooded Lister). S2b: Summary of genes which changed significantly and uni-directionally in response to iron deficiency in both Wistar and Rowett Hooded Lister rats, as analysed by microarray of embryonic tissue. Of 68 genes which satisfied these criteria, 21 were unidentified (not shown). (FC = fold-change, RHL = Rowett Hooded Lister). S2c: Summary of genes which changed significantly and uni-directionally in Wistar rats in response to both protein and iron deficiency, as analysed by microarray of embryonic tissue (FC = fold-change). S2d: Summary of genes which changed significantly and uni-directionally in Rowett Hooded Lister rats in response to both protein and iron deficiency, as analysed by microarray of embryonic tissue. Of 67 genes which satisfied these criteria, 25 were unidentified (not shown). (FC = fold-change) (DOCX) [file pone.0023189.s003.docx]

**Table S2a:**

| **Probe Set ID** | **Gene symbol** | **Gene name** | **Wistar** | | **RHL** | |
| --- | --- | --- | --- | --- | --- | --- |
|  |  |  | **p-value** | **FC** | **p-value** | **FC** |
| 1390594_PM_at | *A2bp1* | ataxin 2-binding protein 1 | 0.04 | 1.10 | 0.04 | 1.11 |
| 1398835_PM_at | *Actb* | Beta-actin | 0.02 | 1.06 | 0.02 | 1.07 |
| 1388243_PM_at | *Gpr176* | Probable G-protein coupled receptor 176 | 0.05 | 0.87 | 0.02 | 0.79 |
| 1384523_PM_at | *Pms1* | protein homolog 1 | 0.04 | 1.11 | 0.03 | 1.12 |
| 1397334_PM_at | *Sec14l1* | SEC14-like protein 1 | 0.04 | 0.93 | 0.01 | 0.83 |

**Table S2b:**

| **Probe Set ID** | **Gene symbol** | **Gene name** | **Wistar** | | **RHL** | |
| --- | --- | --- | --- | --- | --- | --- |
|  |  |  | **p-value** | **FC** | **p-value** | **FC** |
| 1369928_PM_at | *Acta1* | Actin, alpha skeletal muscle | 0.02 | 1.21 | 0.03 | 1.16 |
| 1372624_PM_at | *Ano6* | anoctamin 6 | 0.04 | 1.11 | 0.03 | 1.09 |
| 1390005_PM_at | *Asxl2* |  | 0.04 | 1.06 | 0.02 | 1.09 |
| 1385414_PM_at | *Cd8a* | Cluster of Differentiation 8a | 0.03 | 0.87 | 0.04 | 0.92 |
| 1370235_PM_at | *Dbi* | Diazepam binding inhibitor | 0.02 | 0.85 | 0.04 | 0.92 |
| 1374219_PM_at | *Disp1* | dispatched homolog 1 | 0.04 | 0.84 | 0.04 | 0.88 |
| 1388449_PM_at | *Eef1b2* | Elongation factor 1-beta 2 | 0.04 | 0.96 | 0.04 | 0.98 |
| 1388297_PM_at | *Eef1g* | Elongation factor 1-gamma | 0.01 | 1.04 | 0.02 | 1.05 |
| 1368078_PM_at | *Esm1* | endothelial cell-specific molecule 1 | 0.04 | 0.81 | 0.01 | 0.86 |
| 1374699_PM_at | *Fam84a* | family with sequence similarity 84, member A | 0.04 | 1.27 | 0.01 | 1.17 |
| 1373499_PM_at | *Gas5* | growth arrest-specific 5 | 0.04 | 0.85 | 0.04 | 0.89 |
| 1377577_PM_at | *Gmps* | Guanine monphosphate synthetase | 0.04 | 0.91 | 0.02 | 0.93 |
| 1377624_PM_at | *Gtpbp10* | GTP-binding protein 10 | 0.04 | 0.81 | 0.00 | 0.79 |
| 1398932_PM_at | *Hint1* | histidine triad nucleotide binding protein 1 | 0.03 | 0.93 | 0.01 | 0.96 |
| 1370904_PM_at | *Hla-dma* | major histocompatibility complex, class II, DM alpha | 0.03 | 1.29 | 0.02 | 1.30 |
| 1377182_PM_at | *Itgb3bp* | integrin beta 3 binding protein | 0.03 | 0.78 | 0.01 | 0.84 |
| 1374912_PM_at | *Kif2c* | kinesin family member 2C | 0.04 | 0.91 | 0.05 | 0.93 |
| 1369637_PM_at | *Kif3c* | kinesin family member 3C | 0.01 | 1.22 | 0.02 | 1.19 |
| 1391595_PM_at | *Larp6* | La ribonucleoprotein domain family, member 6 | 0.04 | 1.12 | 0.05 | 1.10 |
| 1397471_PM_at | *Mical2* | microtubule associated monoxygenase, calponin and LIM domain containing 2 | 0.02 | 1.18 | 0.02 | 1.08 |
| 1393085_PM_at | *Mitd1* | microtubule interacting and transport, domain containing 1 | 0.03 | 0.76 | 0.01 | 0.79 |
| 1372808_PM_at | *Mthfd2* | methylenetetrahydrofolate dehydrogenase 2 | 0.05 | 0.88 | 0.02 | 0.93 |
| 1372093_PM_at | *Mxi1* | MAX interactor 1 | 0.04 | 1.11 | 0.04 | 1.09 |
| 1374792_PM_at | *Nol7* | Nucleolar protein 7 | 0.04 | 0.89 | 0.03 | 0.90 |
| 1372172_PM_at | *Nphp1* | nephronophthisis 1 | 0.04 | 0.89 | 0.02 | 0.88 |
| 1388340_PM_at | *Ns5atp9* | NS5A-transactivated protein 9 homolog | 0.02 | 0.88 | 0.01 | 0.91 |
| 1387225_PM_at | *Opa1* | optic atrophy 1 | 0.05 | 0.92 | 0.01 | 0.90 |
| 1380071_PM_at | *Parp12* | poly (ADP-ribose) polymerase 12 | 0.00 | 1.18 | 0.03 | 1.13 |
| 1376025_PM_at | *Prmt2* | protein arginine methyltransferase 2 | 0.05 | 1.13 | 0.03 | 1.21 |
| 1393081_PM_at | *Rasgef1a* | RasGEF domain family, member 1A | 0.04 | 0.89 | 0.02 | 0.90 |
| 1389065_PM_at | *Rbm34* | RNA binding motif protein 34 | 0.01 | 0.92 | 0.05 | 0.87 |
| 1378227_PM_at | *Rbpms2* | RNA binding protein with multiple splicing 2 | 0.01 | 1.10 | 0.01 | 1.14 |
| 1371638_PM_at | *Rnf7* | ring finger protein 7 | 0.05 | 0.92 | 0.04 | 0.94 |
| 1371295_PM_at | *Rps20* | ribosomal protein S20 | 0.05 | 0.94 | 0.03 | 0.94 |
| 1374738_PM_at | *Sdccag10* | serologically defined colon cancer antigen 10 | 0.01 | 0.84 | 0.03 | 0.89 |
| 1370440_PM_at | *Slc15a4* | solute carrier family 15, member 4 | 0.05 | 1.16 | 0.02 | 1.20 |
| 1368991_PM_at | *Smpd3* | sphingomyelin phosphodiesterase 3 | 0.04 | 1.16 | 0.04 | 1.16 |
| 1372167_PM_at | *Snrpg* | small nuclear ribonucleoprotein polypeptide G | 0.02 | 0.87 | 0.01 | 0.89 |
| 1370192_PM_at | *Stx12* | Syntaxin 12 | 0.03 | 1.12 | 0.05 | 1.08 |
| 1371679_PM_at | *Synpo2* | synaptopodin 2 | 0.02 | 1.18 | 0.02 | 1.14 |
| 1374177_PM_at | *Taf13* | TATA box binding protein -associated factor | 0.04 | 0.87 | 0.01 | 0.92 |
| 1390237_PM_at | *Timm8a1* | translocase of inner mitochondrial membrane 8 homolog a1 | 0.01 | 0.82 | 0.01 | 0.88 |
| 1376719_PM_at | *Tmem38b* | transmembrane protein 38B | 0.04 | 0.84 | 0.05 | 0.92 |
| 1398870_PM_at | *Tomm20* | translocase of outer mitochondrial membrane 20 homolog | 0.03 | 0.90 | 0.00 | 0.93 |
| 1370238_PM_at | *Usmg5* | up-regulated during skeletal muscle growth 5 homolog | 0.04 | 0.90 | 0.02 | 0.91 |
| 1371249_PM_at | *Xbp1* | X-box binding protein 1 | 0.01 | 1.18 | 0.04 | 1.27 |
| 1386512_PM_at | *Zfp91* | zinc finger protein 91 homolog | 0.03 | 0.94 | 0.02 | 0.90 |

**Table S2c:**

| **Probe Set ID** | **Gene symbol** | **Gene name** | **Proteins** | | **Irons** | |
| --- | --- | --- | --- | --- | --- | --- |
|  |  |  | **p-value** | **FC** | **p-value** | **FC** |
| 1378310_PM_at | *Nmb* | neuromedin B | 0.03 | 1.13 | 0.02 | 1.11 |
| 1394249_PM_x_at | *Tmem131* | transmembrane protein 131 | 0.01 | 0.91 | 0.02 | 0.92 |

**Table S2d:**

| **Probe Set ID** | **Gene symbol** | **Gene name** | **Proteins** | | **Irons** | |
| --- | --- | --- | --- | --- | --- | --- |
|  |  |  | **p-value** | **FC** | **p-value** | **FC** |
| 1398626_PM_s_at | *Actr2* | actin-related protein 2 homolog | 0.02 | 1.09 | 0.00 | 1.10 |
| 1388179_PM_at | *Acvr2b* | activin A receptor, type IIB | 0.03 | 1.36 | 0.03 | 1.22 |
| 1370510_PM_a_at | *Arntl* | aryl hydrocarbon receptor nuclear translocator-like | 0.01 | 0.84 | 0.02 | 0.87 |
| 1398916_PM_at | *Aurkaip1* | aurora kinase A interacting protein 1 | 0.003 | 0.92 | 0.04 | 0.90 |
| 1368083_PM_at | *Ccnh* | Cyclin H | 0.04 | 0.88 | 0.02 | 0.93 |
| 1382454_PM_at | *Cxcl9* | chemokine (C-X-C motif) ligand 9 | 0.04 | 0.91 | 0.03 | 0.95 |
| 1370311_PM_at | *Eif2b1* | eukaryotic translation initiation factor 2B, subunit 1 alpha | 0.02 | 0.89 | 0.04 | 0.94 |
| 1382169_PM_x_at | *Elavl3* | embryonic lethal, abnormal vision, Drosophila-like 3 | 0.001 | 1.34 | 0.03 | 1.08 |
| 1398903_PM_at | *Esd* | esterase D | 0.01 | 0.92 | 0.00 | 0.90 |
| 1384356_PM_at | *Fam151b* | family with sequence similarity 151, member B | 0.03 | 0.73 | 0.01 | 0.83 |
| 1385072_PM_at | *Galm* | galactose mutarotase | 0.03 | 0.83 | 0.04 | 0.87 |
| 1399098_PM_at | *Glo1* | glyoxalase I | 0.05 | 0.91 | 0.05 | 0.93 |
| 1389918_PM_at | *LOC290704* | similar to palladin | 0.04 | 1.13 | 0.01 | 1.14 |
| 1373232_PM_at | *LOC302022* | similar to nidogen 2 | 0.01 | 0.86 | 0.03 | 0.90 |
| 1386478_PM_at | *Mcart1* | mitochondrial carrier triple repeat 1 | 0.03 | 1.17 | 0.03 | 1.08 |
| 1394005_PM_s_at | *Med21* | mediator complex subunit 21 | 0.02 | 0.75 | 0.03 | 0.88 |
| 1370570_PM_at | *Nrp1* | neuropilin 1 | 0.02 | 1.16 | 0.03 | 1.41 |
| 1394371_PM_at | *Nufip1* | nuclear fragile X mental retardation protein interacting protein 1 | 0.01 | 0.83 | 0.04 | 0.88 |
| 1374571_PM_at | *Pigx* | phosphatidylinositol glycan anchor biosynthesis, class X | 0.05 | 0.91 | 0.01 | 0.89 |
| 1392534_PM_at | *Pmepa1* | prostate transmembrane protein, androgen induced 1 | 0.02 | 1.25 | 0.04 | 1.14 |
| 1381088_PM_at | *Podn* | podocan | 0.01 | 1.16 | 0.05 | 1.12 |
| 1388833_PM_at | *Pole3* | polymerase epsilon 3 | 0.001 | 0.85 | 0.01 | 0.81 |
| 1370536_PM_at | *Prmt3* | protein arginine methyltransferase 3 | 0.02 | 0.86 | 0.001 | 0.87 |
| 1372665_PM_at | *Psat1* | phosphoserine aminotransferase 1 | 0.03 | 0.90 | 0.04 | 0.94 |
| 1367780_PM_at | *Pttg1* | pituitary tumor-transforming 1 | 0.002 | 0.84 | 0.05 | 0.93 |
| 1387911_PM_at | *Rabggtb* | Rab geranylgeranyltransferase, beta subunit | 0.03 | 0.88 | 0.00 | 0.89 |
| 1373461_PM_at | *Rai12* | retinoic acid induced 12 | 0.01 | 0.90 | 0.02 | 0.92 |
| 1385518_PM_at | *RGD1304931* | similar to RIKEN | 0.05 | 0.87 | 0.00 | 0.83 |
| 1377532_PM_at | *RGD1305020* | similar to Hepatocellular carcinoma-associated antigen 58 homolog | 0.01 | 1.21 | 0.04 | 1.12 |
| 1398359_PM_at | *Rnf181* | ring finger protein 181 | 0.02 | 0.73 | 0.02 | 0.80 |
| 1378508_PM_at | *Ssh1* | slingshot homolog 1 | 0.02 | 1.17 | 0.01 | 1.22 |
| 1372077_PM_at | *Strap* | serine/threonine kinase receptor associated protein | 0.02 | 0.94 | 0.04 | 0.95 |
| 1367642_PM_at | *Suclg1* | succinate-CoA ligase, alpha subunit | 0.01 | 0.80 | 0.05 | 0.90 |
| 1377230_PM_at | *Tbx3* | T-box transcription factor 3 | 0.003 | 1.22 | 0.01 | 1.26 |
| 1391405_PM_at | *Thsd7a* | thrombospondin, type I, domain containing 7A | 0.02 | 1.39 | 0.02 | 1.24 |
| 1389471_PM_at | *Tomm34* | translocase of outer mitochondrial membrane 34 | 0.05 | 0.89 | 0.02 | 0.88 |
| 1388557_PM_at | *Tubb2c* | tubulin, beta 2C | 0.05 | 0.90 | 0.04 | 0.91 |
| 1388523_PM_at | *Txndc12* | thioredoxin domain containing 12 | 0.003 | 0.82 | 0.03 | 0.90 |
| 1388484_PM_at | *Ube2c* | ubiquitin-conjugating enzyme E2C | 0.04 | 0.92 | 0.04 | 0.94 |
| 1376086_PM_at | *Ufsp2* | UFM1-specific peptidase 2 | 0.01 | 0.89 | 0.05 | 0.92 |
| 1377961_PM_at | *Zfhx3* | zinc finger homeobox 3 | 0.004 | 1.24 | 0.04 | 1.30 |
| 1372324_PM_at | *Znhit3* | zinc finger, HIT-type containing 3 | 0.01 | 0.88 | 0.03 | 0.90 |
