# Supplementary material for: Cell Cycle Regulation and Cytoskeletal Remodelling Are Critical Processes in the Nutritional Programming of Embryonic Development
Source: PLoS One. 2011 Aug 17;6(8):e23189. doi: 10.1371/journal.pone.0023189 (PMC3157362; doi:10.1371/journal.pone.0023189)
Supplement: Table S3 — Real-time PCR determination of gatekeeper target gene expression in postnatal kidney tissue. (W P = Wistar Proteins, W Fe = Wistar Irons, RHL P = Rowett Hooded Lister Proteins, RHL Fe = Rowett Hooded Lister Irons; FC = fold-change.) (DOCX) [file pone.0023189.s004.docx]

**Table S3:**

| **Gene** | **Group** | **Birth** | | **3 weeks** | | **16 weeks** | |
| --- | --- | --- | --- | --- | --- | --- | --- |
|  |  | **p-value** | **FC** | **p-value** | **FC** | **p-value** | **FC** |
| *TBX3* | W P | 0.21 | 0.72 | 0.001 | 0.35 | 0.75 | 2.68 |
| *HINT1* | W P | 0.35 | 1.55 | 0.14 | 0.58 | 0.34 | 0.77 |
| *eEF1g* | W P | 0.45 | 1.28 | 0.001 | 2.42 | 0.66 | 0.91 |
| *HNF4a* | W P | 0.26 | 0.73 | 0.001 | 0.47 | 0.33 | 0.81 |
| *C-Myc* | W P | 0.05 | 0.62 | 0.001 | 0.07 | 0.35 | 0.83 |
| *SP1* | W P | 0.99 | 1 | 0.15 | 0.45 | 0.08 | 0.71 |
| *Acvr2b* | W P | 0.64 | 0.86 | 0.001 | 0.46 | 0.45 | 1.11 |
| *p53* | W P | 0.1 | 0.59 | 0.001 | 0.42 | 0.67 | 1.29 |
| *pttg1* | W P | 0.63 | 1.25 | 0.001 | 0.34 | 0.35 | 1.37 |
| *stx12* | W P | 0.67 | 1.17 | 0.001 | 0.38 | 0.37 | 1.06 |
| *ccnh* | W P | 0.98 | 1.02 | 0.02 | 0.54 | 0.8 | 1.06 |

| *TBX3* | W Fe | 0.09 | 1.51 | 0.004 | 0.47 | 0.17 | 2.25 |
| --- | --- | --- | --- | --- | --- | --- | --- |
| *HINT1* | W Fe | 0.16 | 2.2 | 0.003 | 0.41 | 0.09 | 1.5 |
| *eEF1g* | W Fe | 0.5 | 1.26 | 0.15 | 0.61 | 0.006 | 2.64 |
| *HNF4a* | W Fe | 0.83 | 0.95 | 0.03 | 0.58 | 0.08 | 2.08 |
| *C-Myc* | W Fe | 0.99 | 1 | 0.06 | 0.53 | 0.03 | 1.11 |
| *SP1* | W Fe | 0.75 | 1.1 | 0.001 | 0.32 | 0.05 | 1.91 |
| *Acvr2b* | W Fe | 0.33 | 0.7 | 0.62 | 0.84 | 0.91 | 2.51 |
| *p53* | W Fe | 0.25 | 0.68 | 0.18 | 0.65 | 0.2 | 2.52 |
| *pttg1* | W Fe | 0.27 | 1.46 | 0.36 | 0.74 | 0.04 | 2.64 |
| *stx12* | W Fe | 0.31 | 0.78 | 0.36 | 0.8 | 0.72 | 2.44 |
| *ccnh* | W Fe | 0.49 | 1.31 | 0.04 | 0.53 | 0.04 | 1.91 |

| *TBX3* | RHL P | 0.02 | 1.88 | 0.02 | 2.24 | 0.02 | 1.78 |
| --- | --- | --- | --- | --- | --- | --- | --- |
| *HINT1* | RHL P | 0.04 | 1.72 | 0.03 | 2.1 | 0.74 | 0.99 |
| *eEF1g* | RHL P | 0.12 | 1.44 | 0.29 | 1.53 | 0.88 | 0.94 |
| *HNF4a* | RHL P | 0.02 | 1.58 | 0.04 | 1.69 | 0.95 | 1.03 |
| *C-Myc* | RHL P | 0.03 | 1.55 | 0.08 | 1.83 | 0.97 | 0.96 |
| *SP1* | RHL P | 0.006 | 1.99 | 0.17 | 2.03 | 0.89 | 1.05 |
| *Acvr2b* | RHL P | 0.29 | 1.34 | 0.64 | 1.12 | 0.08 | 2.3 |
| *p53* | RHL P | 0.2 | 1.56 | 0.46 | 0.81 | 0.23 | 1.27 |
| *pttg1* | RHL P | 0.4 | 1.27 | 0.33 | 1.42 | 0.67 | 1.99 |
| *stx12* | RHL P | 0.28 | 1.37 | 0.53 | 1.19 | 0.06 | 2.06 |
| *ccnh* | RHL P | 0.71 | 0.89 | 0.05 | 2 | 0.001 | 2.76 |

| *TBX3* | RHL Fe | 0.06 | 0.63 | 0.78 | 1.07 | 0.85 | 0.91 |
| --- | --- | --- | --- | --- | --- | --- | --- |
| *HINT1* | RHL Fe | 0.45 | 0.74 | 0.91 | 0.97 | 1 | 0.8 |
| *eEF1g* | RHL Fe | 0.04 | 0.65 | 0.92 | 1.03 | 0.37 | 0.84 |
| *HNF4a* | RHL Fe | 0.02 | 0.61 | 0.82 | 1.05 | 0.66 | 1.11 |
| *C-Myc* | RHL Fe | 0.51 | 0.83 | 0.7 | 0.88 | 0.92 | 1.01 |
| *SP1* | RHL Fe | 0.18 | 0.77 | 0.75 | 0.89 | 0.01 | 1.8 |
| *Acvr2b* | RHL Fe | 0.73 | 0.91 | 0.28 | 1.32 | 0.66 | 0.9 |
| *p53* | RHL Fe | 0.78 | 0.92 | 0.02 | 1.58 | 0.42 | 0.97 |
| *pttg1* | RHL Fe | 0.71 | 1.09 | 0.84 | 1.06 | 0.86 | 1.36 |
| *stx12* | RHL Fe | 0.92 | 1.02 | 0.96 | 0.99 | 0.97 | 0.95 |
| *ccnh* | RHL Fe | 0.14 | 1.46 | 0.86 | 0.95 | 0.19 | 0.78 |
