# Supplementary material for: Cell Cycle Regulation and Cytoskeletal Remodelling Are Critical Processes in the Nutritional Programming of Embryonic Development
Source: PLoS One. 2011 Aug 17;6(8):e23189. doi: 10.1371/journal.pone.0023189 (PMC3157362; doi:10.1371/journal.pone.0023189)
Supplement: Table S4 — Gene expression data for RT2 Profiler Rat Cell Cycle PCR Array. (FC = fold-change, RHL = Rowett Hooded Lister.) (DOCX) [file pone.0023189.s005.docx]

**Table S4:**

| **Unigene** | **GenBank** | **Gene symbol** | **Gene name** | **RHL Protein** | | **RHL Iron** | |
| --- | --- | --- | --- | --- | --- | --- | --- |
|  |  |  |  | **p-value** | **FC** | **p-value** | **FC** |
| Rn.3105 | XM_231137 | *Abl1* | C-abl oncogene 1, receptor tyrosine kinase | 0.15 | 0.64 | 0.96 | 1.01 |
| Rn.79537 | NM_024349 | *Ak1* | Adenylate kinase 1 | 0.13 | 0.64 | 0.66 | 1.06 |
| Rn.19953 | NM_080478 | *Apbb1* | Amyloid beta (A4) precursor protein-binding, family B, member 1 (Fe65) | **0.01** | 0.50 | 0.76 | 1.07 |
| Rn.214048 | XM_236275 | *Atm* | Ataxia telangiectasia mutated homolog (human) | 0.35 | 0.70 | 0.93 | 1.03 |
| Rn.9996 | NM_016993 | *Bcl2* | B-cell leukaemia/lymphoma 2 | 0.94 | 0.94 | 0.50 | 1.11 |
| Rn.48840 | NM_012514 | *Brca1* | Breast cancer 1 | 0.89 | 0.94 | 0.38 | 1.26 |
| Rn.103225 | NM_031542 | *Brca2* | Breast cancer 2 | 0.74 | 0.98 | 0.51 | 0.94 |
| Rn.107499 | NM_012920 | *Camk2a* | Calcium/calmodulin-dependent protein kinase II alpha | 0.79 | 0.90 | 0.25 | 0.78 |
| Rn.9743 | NM_021739 | *Camk2b* | Calcium/calmodulin-dependent protein kinase II beta | 0.39 | 0.60 | 0.16 | 0.65 |
| Rn.10562 | NM_012922 | *Casp3* | Caspase 3, apoptosis related cysteine protease | 0.19 | 0.65 | **0.05** | 1.20 |
| Rn.102823 | NM_001011949 | *Ccna1* | Cyclin A1 | 0.37 | 0.69 | 0.22 | 0.74 |
| Rn.13094 | NM_053702 | *Ccna2* | Cyclin A2 | 0.08 | 0.56 | 0.18 | 1.16 |
| Rn.9232 | NM_171991 | *Ccnb1* | Cyclin B1 | **0.04** | 0.56 | 0.08 | 0.78 |
| Rn.6743 | NM_001009470 | *Ccnb2* | Cyclin B2 | 0.17 | 0.65 | 0.74 | 1.00 |
| Rn.106758 | XM_342812 | *Ccnc* | Cyclin C | 0.15 | 0.65 | 0.53 | 0.94 |
| Rn.22279 | NM_171992 | *Ccnd1* | Cyclin D1 | 0.25 | 0.67 | 0.65 | 1.13 |
| Rn.96083 | NM_022267 | *Ccnd2* | Cyclin D2 | 0.43 | 1.08 | 0.59 | 1.09 |
| Rn.15455 | XM_574426 | *Ccne1* | Cyclin E1 | 0.39 | 0.77 | 0.66 | 1.06 |
| Rn.15126 | XM_340763 | *Ccnf* | Cyclin F | 0.37 | 0.72 | 0.52 | 1.17 |
| Rn.11390 | NM_133571 | *Cdc25a* | Cell division cycle 25 homolog A (S. pombe) | 0.65 | 0.80 | 0.71 | 1.06 |
| Rn.11312 | NM_133572 | *Cdc25b* | Cell division cycle 25 homolog B (S. pombe) | 0.19 | 0.69 | 0.25 | 1.15 |
| Rn.104460 | NM_199501 | *Cdk2* | Cyclin dependent kinase 2 | 0.54 | 0.79 | 0.39 | 1.12 |
| Rn.6115 | NM_053593 | *Cdk4* | Cyclin-dependent kinase 4 | 0.09 | 0.62 | 0.30 | 1.11 |
| Rn.203271 | NM_145721 | *Cdk5rap1* | CDK5 regulatory subunit associated protein 1 | 0.40 | 0.76 | 0.30 | 0.86 |
| Rn.10089 | NM_080782 | *Cdkn1a* | Cyclin-dependent kinase inhibitor 1A (p21, Cip1) | 0.29 | 0.63 | 0.21 | 0.75 |
| Rn.29897 | NM_031762 | *Cdkn1b* | Cyclin-dependent kinase inhibitor 1B | 0.26 | 0.68 | 0.16 | 1.26 |
| Rn.48717 | NM_031550 | *Cdkn2a* | Cyclin-dependent kinase inhibitor 2A | 0.37 | 1.24 | 0.27 | **0.43** |
| Rn.105626 | NM_130812 | *Cdkn2b* | Cyclin-dependent kinase inhibitor 2B (p15, inhibits CDK4) | 0.65 | 0.98 | 0.24 | 0.69 |
| Rn.33267 | NM_080400 | *Chek1* | CHK1 checkpoint homolog (S. pombe) | 0.26 | 0.70 | 0.59 | 0.93 |
| Rn.11183 | NM_024134 | *Ddit3* | DNA-damage inducible transcript 3 | 0.21 | 0.73 | 0.70 | 1.05 |
| Rn.11908 | NM_053776 | *Dnajc2* | DnaJ (Hsp40) homolog, subfamily C, member 2 | 0.28 | 0.67 | 0.78 | 0.98 |
| Rn.79807 | XM_237042 | *Dst* | Dystonin | 0.69 | 1.03 | 0.92 | 1.01 |
| Rn.72471 | XM_230765 | *E2f1* | E2F transcription factor 1 | 0.22 | 0.67 | 0.37 | 1.17 |
| Rn.73967 | XM_214476 | *E2f3* | E2F transcription factor 3 | 0.71 | 0.80 | 0.29 | 1.15 |
| Rn.154586 | XM_226441 | *E2f4* | E2F transcription factor 4 | 0.23 | 0.70 | 0.77 | 1.08 |
| Rn.10250 | NM_024127 | *Gadd45a* | Growth arrest and DNA-damage-inducible, alpha | 0.07 | 0.61 | 0.41 | 0.92 |
| Rn.145095 | XM_234574 | *Gpr132* | G protein-coupled receptor 132 | 0.31 | 1.37 | 0.29 | **0.49** |
| Rn.12812 | XM_573658 | *Hus1* | HUS1 checkpoint homolog (S. pombe) | 0.75 | 0.84 | 0.73 | 0.96 |
| Rn.8831 | NM_012590 | *Inha* | Inhibin alpha | 0.31 | 0.67 | 0.91 | 1.04 |
| Rn.25733 | NM_017022 | *Itgb1* | Integrin beta 1 (fibronectin receptor beta) | 0.10 | 0.67 | 0.69 | 1.11 |
| Rn.9521 | XM_214069 | *LOC289740* | Similar to PES1 protein | 0.39 | 0.74 | 0.46 | 0.91 |
| Rn.8800 | XM_225713 | *LOC307231* | Similar to nuclear factor of activated T-cells, cytoplasmic, calcineurin-dependent 1 | 0.61 | 1.08 | 0.40 | 0.78 |
| Rn.154788 | XM_346381 | *LOC367976* | Similar to DNA replication licensing factor MCM3 (DNA polymerase alpha holoenzyme-associated protein P1) (P1-MCM3) | 0.20 | 0.66 | 0.34 | 0.83 |
| Rn.19771 | XM_216161 | *Mad2l1* | MAD2 (mitotic arrest deficient, homolog)-like 1 (yeast) | 0.81 | 0.81 | 0.98 | 1.02 |
| Rn.2715 | XM_232168 | *Mcm2* | Minichromosome maintenance complex component 2 | 0.18 | 0.70 | 0.73 | 1.05 |
| Rn.8341 | XM_344048 | *Mcm4* | Minichromosome maintenance complex component 4 | 0.19 | 0.69 | 0.58 | 0.92 |
| Rn.91829 | XM_235169 | *Mdm2* | Mdm2 p53 binding protein homolog (mouse) | **0.04** | 0.64 | 0.25 | 1.09 |
| Rn.73551 | XM_225460 | *Mki67* | Antigen identified by monoclonal antibody Ki-67 | 0.98 | 0.89 | 0.55 | 0.94 |
| Rn.209040 | NM_022279 | *Mre11a* | MRE11 meiotic recombination 11 homolog A (S. cerevisiae) | 0.86 | 0.94 | 0.18 | 0.76 |
| Rn.3174 | NM_031058 | *Msh2* | MutS homolog 2 (E. coli) | 0.15 | 0.73 | 0.77 | 1.07 |
| Rn.218571 | XM_344868 | *Nanos2* | Nanos homolog 2 (Drosophila) | 0.24 | 1.33 | 0.19 | **0.31** |
| Rn.144627 | XM_001055166 | *Nek2* | NIMA (never in mitosis gene a)-related expressed kinase 2 | 0.09 | 0.61 | 0.87 | 1.03 |
| Rn.65930 | NM_024358 | *Notch2* | Notch homolog 2 (Drosophila) | 0.58 | 0.83 | 0.88 | 1.04 |
| Rn.214645 | NM_203340 | *Npm2* | Nucleophosmin/nucleoplasmin 2 | 0.68 | 0.76 | 0.30 | 0.58 |
| Rn.223 | NM_022381 | *Pcna* | Proliferating cell nuclear antigen | 0.26 | 0.68 | 0.17 | 0.77 |
| Rn.127770 | XM_340765 | *Pkd1* | Polycystic kidney disease 1 homolog (human) | 0.39 | 0.73 | 0.82 | 1.05 |
| Rn.1476 | NM_017037 | *Pmp22* | Peripheral myelin protein 22 | **0.03** | 0.56 | 0.97 | 1.01 |
| Rn.15540 | XM_213418 | *Ppm1d* | Protein phosphatase 1D magnesium-dependent, delta isoform | 0.20 | 0.68 | **0.03** | 1.24 |
| Rn.93024 | XM_576459 | *Ppp2r3a* | Protein phosphatase 2 (formerly 2A), regulatory subunit B'', alpha | 0.19 | 0.65 | 0.84 | 1.03 |
| Rn.6866 | NM_017041 | *Ppp3ca* | Protein phosphatase 3 (formerly 2B), catalytic subunit, alpha isoform | 0.27 | 0.68 | 0.54 | 1.08 |
| Rn.27657 | NM_001002850 | *Prm1* | Protamine 1 | 0.48 | **0.46** | 0.18 | **0.47** |
| Rn.144759 | XM_214550 | *Psmg2* | Proteasome (prosome, macropain) assembly chaperone 2 | 0.20 | 0.74 | 0.45 | 0.91 |
| Rn.154275 | NM_001024778 | *Rad17* | RAD17 homolog (S. pombe) | **0.05** | 0.65 | 0.81 | 1.03 |
| Rn.3991 | NM_001025701 | *Rad21* | RAD21 homolog (S. pombe) | 0.34 | 0.70 | 0.89 | 1.04 |
| Rn.214052 | NM_001109204 | *Rad51* | RAD51 homolog (RecA homolog, E. coli) (S. cerevisiae) | 0.14 | 0.66 | 0.25 | 1.24 |
| Rn.145016 | XM_219684 | *Rad9* | RAD9 homolog (S. pombe) | 0.06 | 0.64 | 0.92 | 1.02 |
| Rn.107698 | NM_053439 | *Ran* | RAN, member RAS oncogene family | **0.03** | 0.63 | 0.80 | 1.09 |
| Rn.208977 | XM_001055763 | *Rbl1* | Retinoblastoma-like 1 (p107) | 0.45 | 0.75 | 0.77 | 0.94 |
| Rn.11020 | NM_031094 | *Rbl2* | Retinoblastoma-like 2 | 0.21 | 0.68 | 0.80 | 1.07 |
| Rn.216753 | XM_578496 | *RGD1566319* | Similar to Sestrin 2 (Hi95) | 0.20 | 0.70 | 0.11 | 1.25 |
| Rn.145079 | XM_232745 | *Sfn* | Stratifin | 0.85 | 0.72 | 0.50 | 0.93 |
| Rn.138818 | NM_053517 | *Shc1* | SHC (Src homology 2 domain containing) transforming protein 1 | 0.41 | 0.72 | 0.74 | 0.97 |
| Rn.154278 | XM_226817 | *Skp2* | S-phase kinase-associated protein 2 (p45) | 0.19 | 0.69 | 0.49 | 1.07 |
| Rn.211782 | XM_001068751 | *Slfn1* | Schlafen 1 | 0.32 | 1.14 | 0.27 | **0.22** |
| Rn.11763 | NM_031683 | *Smc1a* | Structural maintenance of chromosomes 1A | 0.68 | 0.79 | 0.85 | 1.06 |
| Rn.11715 | XM_236609 | *Stag1* | Stromal antigen 1 | 0.82 | 0.86 | 0.54 | 1.07 |
| Rn.1221 | NM_001009672 | *Sumo1* | SMT3 suppressor of mif two 3 homolog 1 (S. cerevisiae) | 0.15 | 0.68 | 0.78 | 0.95 |
| Rn.144795 | NM_001134735 | *Taf10* | TAF10 RNA polymerase II, TATA box binding protein (TBP)-associated factor | **0.01** | 0.60 | 0.68 | 0.97 |
| Rn.33853 | NM_001012464 | *Terf1* | Telomeric repeat binding factor (NIMA-interacting) 1 | 0.44 | 0.77 | 0.95 | 1.05 |
| Rn.105731 | XM_217232 | *Tfdp2* | Transcription factor Dp-2 (E2F dimerization partner 2) | 0.96 | 0.90 | 0.78 | 0.98 |
| Rn.54443 | NM_030989 | *Tp53* | Tumor protein p53 | 0.11 | 0.61 | 0.68 | 0.97 |
| Rn.42907 | NM_019221 | *Tp73l* | Tumor protein p73-like | 0.67 | 0.79 | 0.57 | 0.89 |
| Rn.7410 | NM_181628 | *Tsg101* | Tumor susceptibility gene 101 | **0.04** | 0.60 | 0.22 | 1.12 |
| Rn.208255 | NM_001012742 | *Wee1* | Wee 1 homolog (S. pombe) | **0.04** | 0.56 | 0.96 | 0.99 |
